# Prevalence and predictors of perceived COVID-19 stigma within a population-based sample of adults with COVID-19

**DOI:** 10.1186/s12889-023-17042-3

**Published:** 2023-10-27

**Authors:** Soomin Ryu, Samhita Chaubal, Paula Guro, Elizabeth J. King, Robert Orellana, Nancy L. Fleischer, Jana L. Hirschtick

**Affiliations:** 1 Department of Epidemiology, School of Public Health, University of Michigan, Ann Arbor, MI 48109 USA; 2 Battelle, Columbus, OH 43201 USA; 3 Defense Centers for Public Health-Portsmouth (DCPH-P), Portsmouth, VA 23708 USA; 4 Department of Health Behavior and Health Education, School of Public Health, University of Michigan, Ann Arbor, MI 48109 USA; 5 CDC Foundation, Atlanta, GA 30308 USA; 6 Michigan Department of Health and Human Services, Lansing, MI 48909 USA

**Keywords:** COVID-19, Stigma, Predictors, Probability survey, United States

## Abstract

**Background:**

Growing evidence suggests that individuals with COVID-19 face stigmatization, which is associated with poor health outcomes and behaviors. However, very few population-based studies have examined risk factors for experiencing COVID-19 stigma. This study examined prevalence and predictors of perceived COVID-19 stigma using a population-based probability sample of adults with COVID-19.

**Methods:**

We included adults with polymerase chain reaction-confirmed SARS-CoV-2 in Michigan between January 1, 2020 and July 31, 2021. Perceived COVID-19 stigma was considered present if a respondent answered affirmatively to any of the following items due to people thinking they might have COVID-19: “you were treated badly,” “people acted as if they were scared of you,” and “you were threatened or harassed.” We conducted modified Poisson regression with robust standard errors to estimate associations between perceived COVID-19 stigma and potential predictors, including sex, age, race and ethnicity, household income, education, employment, smoking status, body mass index, preexisting diagnosed physical or mental comorbidities, and COVID-19 illness severity.

**Results:**

Perceived COVID-19 stigma was commonly reported among our respondents (38.8%, n = 2,759). Compared to those over 65 years, respondents who were 18 − 34 (adjusted prevalence ratio (aPR): 1.41, 95% confidence intervals (CI): 1.12 − 1.77) and 35 − 44 years old (aPR: 1.66, 95% CI: 1.31 − 2.09) reported higher perceived stigma. Female respondents had 1.23 times higher prevalence of perceived COVID-19 stigma (95% CI: 1.10 − 1.37) than male respondents and non-Hispanic Black respondents had 1.22 times higher prevalence of perceived COVID-19 stigma (95% CI: 1.04 − 1.44) than non-Hispanic White respondents. Moreover, respondents with pre-existing diagnosed psychological or psychiatric comorbidities were more likely to report perceived COVID-19 stigma (aPR: 1.29, 95% CI: 1.13 − 1.48) compared to those without diagnosed comorbidities. Respondents with very severe COVID-19 symptoms were also more likely to report perceived COVID-19 stigma (aPR: 1.47, 95% CI: 1.23 − 1.75) than those with asymptomatic or mild symptoms.

**Conclusions:**

We found that populations who are marginalized in United States, such as females, non-Hispanic Black adults, or individuals with chronic conditions, are more likely to report perceived COVID-19 stigma. Continuing to monitor COVID-19 stigma, especially in vulnerable populations, may provide useful insights for anti-stigma campaigns and future pandemics.

## Background

The coronavirus disease 2019 (COVID-19) pandemic has highlighted stigma toward individuals suspected of or diagnosed with COVID-19 [1–5]. In the context of health, stigma occurs when people are labeled, stereotyped, separated, or discriminated against due to a perceived link with a disease [6–8]. Growing evidence suggests that individuals who were diagnosed with COVID-19 faced stigmatization [2], such as being harassed, avoided, and marginalized, including difficulties in service utilization and employment [2–5]. COVID-19 stigma has also affected individuals perceived as associated with the virus [4]. For example, people of Asian descent and healthcare workers suffered from avoidance, rejection, and discrimination [9–11]. This is concerning as stigma can adversely affect multiple life domains, such as educational opportunities, employment, housing, social relationships, health behaviors, and mental health outcomes [7, 12].

Historically, disease outbreaks have been accompanied by stigmatization, leading to adverse outcomes for stigmatized populations [13, 14]. With the human immunodeficiency virus (HIV) epidemic, stigma was reported by individuals who were HIV-positive or disproportionately affected by HIV, such as people from racial and ethnic minoritized groups, lesbian, gay, bisexual, transgender, queer or questioning (LGBTQ) communities, or countries where HIV was endemic [15]. HIV-related stigma is associated with isolation, decreased adherence to HIV treatment, lower usage of health services, and poor self-assessed physical and mental health [15–17]. Similarly, with the 2002 sudden acute respiratory syndrome (SARS) epidemic, individuals with SARS or of Asian origin suffered from social avoidance, verbal harassment, and physical abuse [18, 19]. Furthermore, perceived stigma was associated with an increased risk of psychiatric comorbidities among individuals with SARS infection [20]. In addition, during the 2014–2016 West Africa Ebola outbreak, individuals with Ebola or from African countries faced high levels of stigmatization, which was a risk factor for psychological disorders [21–24].

A few recent studies have examined COVID-19 stigma [8, 11, 25, 26]. One small study of Chinese adults found that individuals who had COVID-19, had family members with COVID-19, or had other social stressors were likely to report greater stigma [11]. A small study from Qatar of individuals with COVID-19 suggested that manual laborers and respondents with lower education reported higher levels of perceived COVID-19 stigma [25]. According to a U.S.-based online survey, being Asian, working as a healthcare worker or first responder, being at high risk of serious illness, and having COVID-19 or COVID-like symptoms predicted COVID-19 stigma [8]. Another nationally representative U.S. study found that individuals who were non-Hispanic Black, Asian, and who wore face masks were more likely to experience COVID-19 stigma compared to their counterparts [26].

To date, we are only aware of one U.S. study using a probability sample to explore COVID-19 stigma [26]. A limitation to this study’s findings was that it did not examine a comprehensive set of factors, such as pre-existing health conditions or COVID-19 illness-related variables that may be associated with COVID stigma. This substantial lack of population-based studies limits our understanding of the prevalence and risk factors of COVID-19 stigma, which is crucial given the emergence of COVID-19 stigmatization and the lasting negative effects of stigma on mental health and well-being [1–5, 8]. The current study fills this gap in the literature by using a population-based probability survey of adults with COVID-19 onset in Michigan between January 1, 2020 and July 31, 2021 to examine the prevalence and potential risk factors for perceived COVID-19 stigma, including demographic and socioeconomic characteristics, pre-existing conditions, health behavior, and COVID-19 illness severity.

## Methods

### Data

We used cross-sectional data from the Michigan COVID-19 Recovery Surveillance Study (MI CReSS), a population-based probability sample of adults with a positive polymerase chain reaction (PCR) SARS-CoV-2 test. Eligibility criteria for participation in the study included: (1) noninstitutionalized and aged 18 years old or older living in Michigan, (2) PCR-confirmed SARS-CoV-2 infection recorded in the Michigan Disease Surveillance System (MDSS), (3) valid phone number and geographic information (county/zip code), and (4) alive at the time of the sample draw. All respondents were invited to participate in the study via mailed letters, and took the survey either (1) on the phone with a trained interviewer in English, Spanish, or Arabic, or (2) online in English.

Individuals were sampled based on their timing of COVID-19 onset and geographic strata, which included 6 counties (Macomb, Oakland, Saint Clair, Monroe, Washtenaw, and Wayne [except Detroit]), 6 public health preparedness regions [27], and the city of Detroit. The timing of COVID-19 onset was determined based on self-reported symptom onset date (if available), the collection date of the positive SAR-CoV-2 test (if available), or the referral date to the Michigan Department of Health and Human Services (MDHHS). This study included individuals with illness onset, test collection, or referral dates between January 2020 and July 2021. Respondents completed surveys between June 2020 and April 2022. To account for nonresponse, we applied sampling weights using generalized regression estimators [28] to match the weighted distribution of our sample to the age and sex distribution of the sampling frame. The median time from onset, test collection, or referral to survey completion was 22 weeks (interquartile range [IQR]: 18 − 27 weeks), and the response rate was 32.1% (American Association for Public Opinion Research response rate #6) [29].

For this analysis, we excluded respondents with missing information on variables of interest, except for missing household income, which was imputed using the weighted sequential hot deck method [30] and hot deck propensity score imputation [31]. Of the 11,000 adults selected in the 10 MI CReSS waves, 3,434 completed the survey. Our analysis excluded 398 respondents who were surveyed before the perceived COVID-19 stigma questions were added to the survey tool and 263 respondents with missing information for perceived COVID-19 stigma or predictor variables of interest. An additional 14 surveys collected via proxy respondents due to mental capacity issues (n = 9) or some other reason rendering them unable to complete the survey (n = 5) were excluded from the analysis. Thus, our analytic sample was n = 2,759. The University of Michigan institutional review board deemed this study exempt due to the use of secondary de-identified data and all respondents provided consent to participate.

### Outcome variable

We assessed perceived COVID-19 stigma using a version of the Everyday Discrimination Scale [32] that was previously adapted for use during the COVID-19 pandemic [33]. We asked whether respondents experienced any of the following things due to people thinking they might have COVID-19: “you were treated badly or without respect,” “people acted as if they were scared of you,” and “you were threatened or harassed.” We created a binary variable of perceived COVID-19 stigma, which equaled 1 if the respondent answered affirmatively to any of the three items and 0 otherwise [26, 34].

### Potential predictors

Potential predictors of perceived COVID-19 stigma were demographic and socioeconomic characteristics: age (18 − 34, 35 − 44, 45 − 54, 55 − 64, ≥ 65), sex (male, female), race and ethnicity (Hispanic, non-Hispanic White, non-Hispanic Black, another non-Hispanic race and ethnicity), marital status (married/cohabitating, not currently married/cohabitating), household income (<$35,000, $35,000 − 74,999, ≥$75,000), and employment (healthcare worker, non-healthcare worker, unemployed [out of work, homemaker, student, retired, unable to work]). We also included variables related to health status and behaviors, such as cigarette smoking status (smoked at least 100 cigarettes in lifetime and every day or some days immediately prior to illness), Body Mass Index (BMI; underweight/normal weight [< 25], overweight [≥ 25 and < 30], obese [≥ 30]), having any pre-existing diagnosed physical comorbidities (chronic obstructive pulmonary disease [COPD], asthma, diabetes, cardiovascular disease, high blood pressure, liver disease, kidney disease, cerebrovascular disease, cancer, immunosuppressive condition, autoimmune condition, or physical disability), and a pre-existing diagnosed psychological or psychiatric condition. Additionally, we examined self-reported COVID-19 symptom severity (asymptomatic/mild, moderate, severe, very severe) and hospitalization for COVID-19 (yes, no).

### Statistical analysis

We first conducted a descriptive analysis of study variables for the entire analytic sample and by perceived COVID-19 stigma status. To compare differences between subgroups, we conducted χ^2^ tests for categorical variables. Then, we conducted modified Poisson regression with robust standard errors to estimate prevalence ratios for associations between the independent variables and perceived COVID-19 stigma. We conducted four regression models, which sequentially added blocks of potential predictors including demographic characteristics (age, sex, race and ethnicity, marital status), socioeconomic variables (employment status, household income), health status and behaviors (smoking status, BMI, pre-existing diagnosed physical comorbidities, pre-existing diagnosed psychological or psychiatric condition), and COVID-19 severity (self-reported COVID-19 symptom severity, hospitalization for COVID-19). All models adjusted for survey mode (phone or online) and date of COVID-19 onset. We conducted statistical analyses using Stata, version 17 and incorporated sampling strata and survey weights for all analyses.

## Results

Table 1 presents the distribution of study variables for the sample overall and stratified by perceived COVID-19 stigma. A total of 1,050 (38.8%) respondents perceived COVID-19 stigma. According to the χ^2^ tests, there was a statistically significant difference between individuals who did or did not report perceived stigma by age group, sex, race and ethnicity, marital status, employment status, household income, pre-existing psychological or psychiatric diagnosis, and COVID-19 symptom severity. Compared to respondents who did not report perceived COVID-19 stigma, respondents who reported perceived stigma were more likely to be younger, female, non-White, not married or cohabiting, employed, and to have lower income, a pre-existing psychological or psychiatric condition, and more severe COVID-19 symptomatology.

**Table 1:** Weighted prevalence of characteristics of study participants, Michigan COVID-19 recovery surveillance study, 2020–2021 (n = 2,759)

		COVID-19 stigma		
	Total	Yes	No	p-value
	(100%)	(n = 1,050, 38.8%)	(n = 1,709, 61.2%)	
Age group				0.000
18–34	37.4	39.6	35.9	
35–44	17.1	20.5	15.0	
45–54	17.5	17.0	17.8	
55–64	15.9	14.5	16.8	
≥65	12.1	8.4	14.5	
Sex				0.000
Male	46.1	39.9	50.0	
Female	53.9	60.1	50.0	
Race and ethnicity				0.017
Hispanic	8.6	9.4	8.1	
Non-Hispanic White	71.5	67.9	73.7	
Non-Hispanic Black	9.5	11.5	8.3	
Another non-Hispanic race and ethnicity	10.4	11.2	9.9	
Marital status				0.022
Married or cohabiting	62.1	59.2	64.0	
Not married or cohabiting	37.9	40.8	36.0	
Employment status				0.021
Healthcare worker	12.1	13.7	11.2	
Non-healthcare worker	60.2	61.5	59.4	
Unemployed	27.7	24.8	29.5	
Household income				0.010
<$35,000	30.9	33.8	29.0	
$35,000–74,999	30.7	31.4	30.2	
≥$75,000	38.4	34.8	40.8	
Pre-existing diagnosed physical comorbidities				0.403
Yes	49.0	50.1	48.3	
No	51.0	49.9	51.7	
Pre-existing diagnosed psychological or psychiatric condition				0.000
Yes	12.9	16.6	10.5	
No	87.1	83.4	89.5	
Body mass index				0.179
Underweight/normal	26.3	28.4	24.9	
Overweight	31.4	30.9	31.7	
Obese	42.3	40.7	43.4	
Cigarette smoking directly prior to COVID-19 illness				0.606
Yes	8.6	9.0	8.4	
No	91.4	91.0	91.6	
COVID-19 symptom severity				0.000
Asymptomatic/mild	32.4	26.6	36.0	
Moderate	32.4	32.6	32.2	
Severe	23.5	26.5	21.6	
Very severe	11.7	14.3	10.2	
Hospitalized				0.859
Yes	9.1	9.3	9.1	
No	90.9	90.7	90.9	

*Note* Weighted percentages are reported

COVID-19, coronavirus disease 2019

Table 2 reports regression results for a series of models estimating associations between potential predictors and perceived COVID-19 stigma. The adjusted prevalence ratios (aPR) and 95% confidence intervals (CI) are reported. Across all models, age, sex, and race and ethnicity were statistically significant predictors, while marital status was not. When we included only demographic characteristics (Model 1), all age groups under 65 had a higher prevalence of perceived COVID-19 stigma than individuals aged 65 or older; the relative difference was most pronounced for adults aged 35 to 44 (aPR: 1.69, 95% CI: 1.37 − 2.08). Female respondents had 1.26 times higher prevalence of perceived COVID-19 stigma (95% CI: 1.13 − 1.41) than male respondents, while non-Hispanic Black respondents had 1.22 times higher prevalence of perceived COVID-19 stigma (95% CI: 1.04 − 1.42) than non-Hispanic White respondents. The magnitude and statistical significance of these associations remained when we included socioeconomic, health status, and behavior variables in Models 2 and 3. When COVID-severity measures were additionally included in Model 4, the prevalence of perceived COVID-19 stigma was still higher among younger individuals aged 18 to 34 (aPR: 1.41, 95% CI: 1.12 − 1.77), 35 to 44 (aPR: 1.66, 95% CI: 1.31 − 2.09), and 45 to 54 (aPR: 1.30, 95% CI: 1.02 − 1.65) compared to individuals aged 65 or older. Female respondents had 1.23 times higher prevalence of perceived COVID-19 stigma (95% CI: 1.10 − 1.37) than male respondents and non-Hispanic Black respondents had 1.22 times higher prevalence of perceived COVID-19 stigma (95% CI: 1.04 − 1.44) than non-Hispanic White respondents. Respondents who were Hispanic and another non-Hispanic race or ethnicity also had a slightly higher prevalence of perceived COVID-19 stigma than non-Hispanic White respondents, although these associations were not statistically significant.

**Table 2:** Prevalence Ratio for Associations between Study Variables and COVID-19 Stigma, Michigan COVID-19 Recovery Surveillance Study, 2020–2021 (n = 2,759)

Dependent variable: Reporting COVID-19 stigma	Model 1 aPR (95% CI)	Model 2 aPR (95% CI)	Model 3 aPR (95% CI)	Model 4 aPR (95% CI)
Age group (ref: ≥65)				
18–34	1.48***	1.41**	1.42**	1.41**
	(1.22–1.81)	(1.14–1.75)	(1.13–1.78)	(1.12–1.77)
35–44	1.69***	1.65***	1.68***	1.66***
	(1.37–2.08)	(1.32–2.07)	(1.33–2.11)	(1.31–2.09)
45–54	1.36**	1.34*	1.34*	1.30*
	(1.10–1.69)	(1.06–1.69)	(1.06–1.69)	(1.02–1.65)
55–64	1.31*	1.29*	1.29*	1.26†
	(1.05–1.63)	(1.02–1.63)	(1.02–1.63)	(1.00–1.59)
Female (ref: male)	1.26***	1.26***	1.24***	1.23***
	(1.13–1.41)	(1.13–1.41)	(1.11–1.38)	(1.10–1.37)
Race and ethnicity (ref: non-Hispanic White)				
Hispanic	1.08	1.03	1.08	1.05
	(0.90–1.30)	(0.86–1.24)	(0.90–1.29)	(0.87–1.26)
Non-Hispanic Black	1.22*	1.17†	1.23*	1.22*
	(1.04–1.42)	(1.00–1.38)	(1.04–1.45)	(1.04–1.44)
Another non-Hispanic race and ethnicity	1.07	1.06	1.09	1.07
	(0.91–1.27)	(0.89–1.25)	(0.92–1.29)	(0.91–1.27)
Married or cohabiting (ref: not married or cohabiting)		0.97 (0.86–1.08)	0.97 (0.87–1.09)	0.97 (0.87–1.08)
Employment status (ref: unemployed)				
Healthcare worker		1.03	1.06	1.05
		(0.86–1.23)	(0.89–1.27)	(0.88–1.26)
Non-healthcare worker		1.10	1.15*	1.14†
		(0.96–1.26)	(1.01–1.32)	(1.00–1.30)
Household income (ref: ≥$75,000)				
<$35,000		1.16*	1.14†	1.11
		(1.01–1.33)	(0.99–1.30)	(0.96–1.27)
$35,000–74,999		1.13†	1.14†	1.11
		(0.99–1.29)	(1.00–1.29)	(0.98–1.26)
Pre-existing diagnosed physical comorbidities (ref: no)			1.16** (1.04–1.30)	1.13* (1.01–1.27)
Pre-existing diagnosed psychological or psychiatric condition (ref: no)			1.31*** (1.15–1.50)	1.29*** (1.13–1.48)
Body mass index (ref: underweight/normal)				
Overweight			0.91	0.90
			(0.80–1.04)	(0.79–1.03)
Obese			0.84**	0.81**
			(0.73–0.95)	(0.71–0.93)
Cigarette smoking directly prior to COVID-19 illness (ref: no smoking)			0.98 (0.83–1.16)	0.99 (0.84–1.18)
COVID-19 symptom severity (ref: asymptomatic/mild)				
Moderate				1.21**
				(1.05–1.38)
Severe				1.34***
				(1.16–1.54)
Very severe				1.47***
				(1.23–1.75)
Hospitalized (ref: no)				0.99
				(0.82–1.20)

*Note* All regression models included survey mode and date of COVID-19 onset. †p < 0.1, * p < 0.05, ** p < 0.01, *** p < 0.001

COVID-19, coronavirus disease 2019; aPR, adjusted prevalence ratio; CI, confidence interval

Socioeconomic factors were less strongly associated with the prevalence of perceived COVID-19 stigma than demographic characteristics. Compared to adults who were unemployed, adults employed in non-healthcare settings were more likely to perceive COVID-19 stigma, although this association was only significant after accounting for health status (Model 3). Additionally, adults with a lower household income were more likely to perceive COVID-19 stigma than adults with a household income of $75,000 or more, although results were not statistically significant after adjusting for health status and behaviors (Model 3) and COVID-19 severity (Model 4).

Several health status variables were associated with perceived COVID-19 stigma prevalence. Respondents with pre-existing diagnosed physical comorbidities had 1.16 times higher prevalence of perceived COVID-19 stigma (95% CI: 1.04 − 1.30) than respondents without pre-existing diagnosed physical comorbidities (Model 3); the association remained after the addition of COVID-19 severity measures (Model 4). Moreover, respondents who had a pre-existing diagnosed psychological or psychiatric condition had 1.31 times higher prevalence of perceived COVID-19 stigma (95% CI: 1.15 − 1.50) than respondents who did not have a psychological or psychiatric condition (Model 3); the association remained in Model 4. We also found that respondents who were obese had lower prevalence of perceived COVID-19 stigma than those who were underweight or normal weight (aPR: 0.84; 95% CI: 0.73 − 0.95 in Model 3; aPR: 0.81, 95% CI: 0.71 − 0.93 in Model 4).

COVID-19 symptom severity was significantly associated with perceived COVID-19 stigma. The prevalence of perceived COVID-19 stigma was higher among individuals who reported moderate (aPR: 1.21, 95% CI: 1.05 − 1.38), severe (aPR: 1.34, 95% CI: 1.16 − 1.54), or very severe (aPR: 1.47, 95% CI: 1.23 − 1.75) COVID-19 symptoms compared to those who reported asymptomatic or mild COVID-19 symptoms (Model 4). On the contrary, hospitalization for COVID-19 was not statistically associated with perceived COVID-19 stigma prevalence (aPR: 0.99, 95% CI: 0.82 − 1.20).

## Discussion

The current study contributes to the literature by using a population-based sample to estimate the prevalence and predictors of perceived COVID-19 stigma among Michigan adults with confirmed SAR-CoV-2 infection. We found that more than one-third of adults with confirmed SARS-CoV-2 infections reported perceived COVID-19 stigma. Moreover, our findings suggest that adults who were younger, female, or non-Hispanic Black were more likely to report perceived COVID-19 stigma than adults who were older, male, or non-Hispanic White, respectively. Additionally, adults who had pre-existing chronic conditions and reported more severe acute COVID-19 symptoms were more likely to report perceived COVID-19 stigma than adults who did not have pre-existing conditions or had asymptomatic or mild COVID-19 symptoms, respectively. Individuals whose BMI was classified as obese were less likely to report perceived COVID-19 stigma than individuals classified as underweight or normal weight in models adjusting for other demographic, socioeconomic, and health status factors.

Our findings for sociodemographic factors predicting perceived COVID-19 stigma are consistent with prior research. For age, a prior study among patients with COVID-19 in China also demonstrated that younger age was associated with reporting more COVID-19 stigma [35]. This may be because younger individuals have several social roles, responsibilities, and activities for supporting family members and they worry about the effects of COVID-19 infection on their life, career, and family [35]. With regard to sex, our finding is consistent with a study among patients with COVID-19 in India, which found that female respondents reported higher levels of COVID-19 stigma than males [36]. For race and ethnicity, our finding is aligned with several studies that suggested Black populations were more likely to suffer from COVID-19 stigma than their White counterparts, possibly due to long-standing structural racism and discrimination [26, 37, 38]. Relatedly, the higher prevalence of COVID-19 stigma among individuals who were female or non-Hispanic Black may be because the stigma of COVID-19 interacts with other forms of stigma related to minoritized social identities, such as sex and race [39–41]. This intersectionality due to multiple stigmatized identities can intensify people’s experiences with COVID-19 stigma [39, 40].

Moreover, our finding that COVID-19 symptom severity was associated with perceived COVID-19 stigma is consistent with a previous study. A U.S. study using a convenience sample reported that individuals who had COVID-19 symptoms were more likely to experience COVID-19 stigma compared to those who did not have COVID-19 symptoms [8]. A similar phenomenon was also documented with HIV, in which reduced HIV symptom burden was associated with reduced HIV-related stigma [42, 43].

This study extends the literature by using a probability sample and a comprehensive set of predictors to provide more accurate information on the prevalence and risk factors of COVID-19 stigma in the U.S. Moreover, we newly documented that pre-existing physical or psychological/psychiatric conditions were independently associated with perceived COVID-19 stigma. Previous studies demonstrated that individuals with chronic diseases such as diabetes [44], cancer [45], physical disabilities [46], and psychiatric illness [47] had disease-related stigma, such as being rejected and discriminated against. Thus, existing disease stigma among individuals with pre-existing diagnosed conditions might be exacerbated after COVID-19 illness. Interestingly, our results also suggest that respondents who were obese were less likely to report perceived COVID-19 stigma than those who were underweight or normal weight. Although the mechanism underlying this relationship is not clear, this may be because populations who were obese had existing stigma related to their weight [47] so they might be less sensitive to another disease-related stigma. Additionally, there could be selection bias in that individuals who were obese might not be able to participate in the survey because obesity is a crucial risk factor for severity of COVID-19 illness such as hospitalization, intensive care unit (ICU) admission, and mortality [48, 49]. Further research is needed to jointly examine weight stigma and perceived COVID-19 stigma during the pandemic.

Our findings suggest that adults who are younger, female, non-Hispanic Black, have pre-existing chronic conditions, or have severe COVID-19 symptoms are at greater risk of perceived COVID-19 stigma than their counterparts. According to a previous study, individuals who have disease stigma are likely to suffer from persistent psychiatric morbidities in the long term [20]. To understand potential adverse health effects, there is a need to monitor the trends and effects of COVID-19 stigma, particularly among populations at greatest risk. Governments and communities should consider providing anti-stigma campaigns and educational programs to improve awareness of COVID-19 stigma by emphasizing adverse consequences and addressing negative stereotypes that may lead to stigma.

This study has several limitations. First, with cross-sectional data, we were not able to determine causation in the associations between predictors and perceived COVID-19 stigma. Second, the sample may have recall bias as respondents participated in interviews weeks after their COVID-19 onset, with a median response time of 22 weeks. Third, our sample included adults with a PCR-confirmed SARS-CoV-2 that was recorded in the MDSS and who were alive at the time of survey. The sample may have selection bias because individuals could not participate in our survey if they had COVID-19 but did not get a PCR test due to lack of access to COVID-19 testing [50, 51], fear of COVID-19 stigma [52], or severe COVID-19 illness resulting in death, which may limit generalizability. Fourth, selection bias is possible given our survey response rate of 32.1%; however, the sampling weights were adjusted to match the age and sex distribution of our sampling frame to account for nonresponse, which help to minimize this bias. Finally, we were unable to disaggregate individuals classified as “another non-Hispanic race or ethnicity” due to the small sample size of some of the racial and ethnic subgroups. As growing evidence has shown that Asian U.S. adults severely suffered from discrimination due to COVID-19 [8, 53–55], disaggregated analysis could have provided richer information. Future studies on this topic should prospectively examine predictors of COVID-19 stigma using a sample of larger and more diverse racial and ethnic groups to provide more accurate longitudinal associations and heterogeneity by racial and ethnic group.

## Conclusions

The current study provides new information on the prevalence and predictors of perceived COVID-19 stigma using a population-based probability sample of adults with confirmed COVID-19, with crucial public health implications given the lasting adverse effects of stigma on mental health. Our findings suggest that populations who are marginalized in society such as females, non-Hispanic Black adults, or individuals with chronic conditions, are more likely to suffer from perceived COVID-19 stigma. Although COVID-19 stigma may be evolving as SARS-CoV-2 infections become increasingly widespread, it is critical to understand the risk factors and effects of COVID-19 stigma and apply lessons learned to future pandemics.

## Data Availability

Although the dataset used in this study is not currently available to others, we are in the process of making a de-identified dataset and data dictionary available. Any requests for the data in the interim can be sent to the MI CReSS study team at michigan-cress@umich.edu.
